# The thickness changes of retina in high myopia patients during the third trimester of pregnancy: a pilot study

**DOI:** 10.1186/s12886-021-02137-5

**Published:** 2021-10-27

**Authors:** Chenchen Liu, Puying Wei, Jun Li

**Affiliations:** Department of Ophthalmology, Peking University First Hospital, No.8 Xishiku Street, Xicheng District, Beijing, 100034 China

**Keywords:** High myopia, Pregnancy, Optical coherence tomography, Retinal thickness

## Abstract

**Objectives:**

To observe and compare the difference in retinal thickness using optical coherence tomography (OCT) between patients with high myopia (HM) during the third trimester of pregnancy and age-matched HM non-pregnant women.

**Methods:**

A case-control study. A total of 39 eyes from 39 HM women in the third trimester (study group) and 50 eyes of 50 age-matched non-pregnant women with HM (control group) were included. All subjects underwent SD-OCT examination. The built-in software was used to measure the retinal thickness in macular region. The data from two groups were compared using independent-samples *t* test.

**Results:**

Among the 89 subjects in this study, the mean gestational age of the study group was 35.09 ± 2.44 weeks, and the average age was 32.24 ± 3.75 years. The average age of the control group was 34.04 ± 7.19 years old. Compared with the control group, the average thickness of parafoveal area, and the average thickness of parafoveal superior, inferior, temporal quadrants of the superficial retina and the average thickness of the foveal and parafoveal of the superficial retina were significantly decreased in the study group (*P* < 0.05). Compared with the control group, the average thickness of all quadrants of the retina in the parafoveal area except the nasal quadrant were significantly decreased in the study group (*P* < 0.05).

**Conclusions:**

In this observational study, the retinal thickness of patients with high myopia during the third trimester of pregnancy was thinner than that of non-pregnant women with age-matched high myopia.

## Background

HM is one of the causes of severe visual impairment leading to the major global public health concern [1]. In recent decades, the prevalence of myopia has increased worldwide, especially in Asia. It has reported that in 2012, among the population over 5 years old in China, the total number of myopic patients was about 450 million, of which the HM patients reached 30 million [2]. For the Asian population, it is estimated that the number of myopia among people aged between 25 and 40 will be as high as 78.8% by 2050 [1].

HM, which is defined as a myopic refractive error of ≥6 diopters (D) or an axial length (AL) of > 26.5 mm, is accompanied by characteristic pathological changes, including leopard fundus, conus, macular lesions, peripheral retinal and choroidal lesions [1]. The physiological ophthalmic changes during pregnancy mainly include decreased corneal sensitivity, increased corneal thickness and curvature and decreased intraocular pressure [2, 3]. Due to the significant changes in hormone levels and hemodynamics during pregnancy, the choroid is the structure with the most abundant blood vessels in the eye. It accounts for more than 70% of the total blood flow in the eye [4]. Therefore, it is speculated that the choroid and retina are susceptible to pregnancy. HM is associated with retinal changes, and HM patients is a high-risk group of retinal detachment. It is of great significance to understand the ocular changes for better care of HM pregnant women in the third trimester of pregnancy.

Due to the relative difficulty of sample collection and the limitations of traditional examination methods, the effect of pregnancy on the retina of HM patients is still remain unclear. With the development of OCT technology, a feasible and safe method is provided for quantitative observation of fundus changes during pregnancy in vivo. So using OCT to observe the structure of each retinal layer and measure parameters such as retinal thickness can provide a reference for the evaluation of fundus changes in pregnant women with HM [5].

Considering the safety of mothers and fetus and the fact that the hormone levels and hemodynamic changes in pregnant women may be more obvious in the third trimester of pregnancy, the uninvasive OCT technology has been applied in this study to quantify the superficial, deep and full retinal thickness of the macular region in women with HM in the third trimester of pregnancy. The aim of this study is to observe the corresponding fundus changes of HM during this special period of pregnancy, and try to provide a pilot understanding for further research on the correlation between the retina and pregnancy in the future.

## Subjects and methods

This study is a case-control study. HM women in the third trimester of pregnancy and HM non-pregnant women who conformed to the criteria of ophthalmic examination in Ophthalmology Department of Peking University First Hospital from November 2020 were recruited. This study was approved by the Ethics Committee of Peking University First Hospital and complied with the Declaration of Helsinki. All subjects signed the informed consent form.

Inclusion criteria included age 25–40 years subjects who is generally in good health and has no history of significant cardiovascular and cerebrovascular diseases. Their myopic refractive error ≥ 6 D (or the AL > 26.5 mm) and gestational age is over 28 weeks. The right eye was designated the study eye with an even-numbered birth month; the left eye, for those with an odd-numbered birth month.

Exclusion criteria comprised previous history of hypertension, kidney disease, cardiovascular disease and other systemic diseases. Subjects with a family history of glaucoma, genetic-related eye diseases, history of eye diseases such as glaucoma, cataract, vitreoretinal disease, eye surgery and other ophthalmic diseases were excluded. Subjects with posterior complications of HM, such as retinal schesis, retinal detachment etc. were excluded. Subjects with pregnancy-induced complications (e.g., pre-eclampsia [6]) were excluded. Subjects who are unable to complete the examination and obtain clear fundus images due to various reasons were excluded. The subjects with visual acuity is less than 20/200 were also excluded.

The subjects were divided into two groups: the study group consisted of 39 eyes of 39 HM women in the third trimester of pregnancy and the control group had 50 eyes of non-pregnant women with HM.

All participants underwent standard ophthalmologic examinations, including slit-lamp biomicroscopy, refraction and best corrected visual acuity (BCVA), intraocular pressure (IOP) testing, dilated fundus examination, AL measurement (IOL Master500; Carl Zeiss Meditec, Dublin, CA). BCVA is converted to logarithm of the minimum angle of resolution (logMAR) for analyzing the results.

SD-OCT auxiliary examination had been operated for all subjects by the same skilled examiner (HRA/Spectralis, Heidelberg Engineering, Germany). Linear scanning at the same retinal position is to obtain > 90 images for real-time noise reduction. A total of 31 high-resolution raster lines were scanned in the macula region at 30° × 15°. The horizontal lines were separated by 240 μm. The image quality is checked by the same reader. The inner boundary of the superficial retina is the internal limiting membrane (ILM), and the outer boundary is the approximate form of the inner plexiform layer (IPL). It is equivalent to the interface between IPL and the inner nuclear layer (INL) [7]. The full retinal thickness refers to the thickness from the ILM to the retinal pigment epithelium (RPE) layer. The deep retinal thickness is the thickness from the junction of IPL and INL to RPE. Using the built-in software in the system to measure the superficial, deep and full retinal thickness of the macular region. Foveal thickness at 1 mm and parafoveal thickness at 3 mm diameter were measured by OCT. Parafoveal thickness was recorded for upper, temporal, inferior and nasal quadrants. Three measurements were taken from the retina for each eye. The average of three measurements was used in the study.

SPSS statistical software (IBM SPSS Corporation, version 23.0) was used for statistical analysis. The data were tested for normality and homogeneity of variance. Normal distribution of measurement data were represented by mean ± SD, comparison between the two groups use independent sample t-test; The measurement data of non-normal distribution were represented by M (P_25_, P_75_), and the comparison between the two groups was performed by Mann-Whitney U test. Chi-square test was used to compare categorical variables between the two groups. A *P* value of <0.05 was considered statistically significant.

## Results

### Clinical characteristics

A total of 89 eyes of 89 subjects were included in this study, including 39 study eyes and 50 control eyes. Mean gestational age of the study group was 35.09 ± 2.44 weeks. The clinical characteristics of subjects are shown in Table 1. The age, IOP, AL, diopter(D) and LogMAR visual acuity of the study eyes were compared with the control eyes. The difference was not statistically significant (P>0.05).

**Table 1:** Clinical characteristics of the subjects

Characteristic	Study eyes *n* = 39	Control eyes *N* = 50	P value
	mean ± SD	mean ± SD	
Age (years)	32.24 ± 3.75	34.04 ± 7.19	0.132
IOP (mmHg)	15.03 ± 2.81	15.85 ± 2.22	0.208
Axial length (mm)	26.86 ± 1.02	26.57 ± 1.19	0.286
Diopter(D)	−8.40 ± 1.36	−8.27 ± 1.92	0.274
LogMAR	0	0	1.000

IOP, intraocular pressure; LogMAR, logarithm of the minimum angle of resolution; SD, standard deviation. P < 0.05, independent sample t-test

### Retinal thickness measurements

#### Superficial retinal thickness measurements

**Fig. 1:**
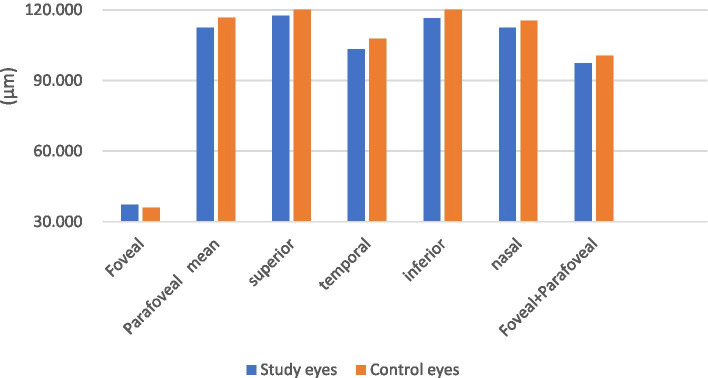
Superficial retinal thickness

**Fig. 2:**
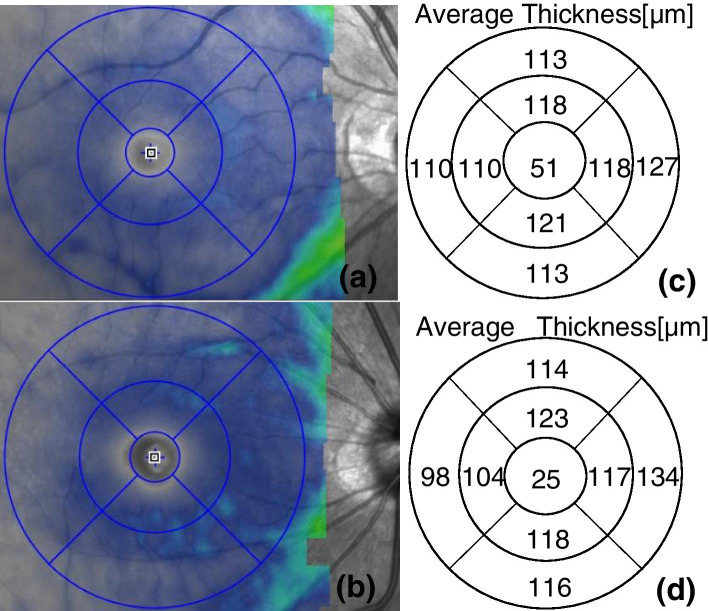
Superficial retinal thickness of an study eye (**a**) and a control eye (**b**). (**c**) and (**d**) are the results of each quadrant thickness of superficial retina automatically measured by the system software (the numbers in the corresponding area in the figure) respectively

The results of superficial retinal thickness in macular region of the study eyes and control eyes are shown in Table 2 and Fig. 1. The representative OCT images are shown in Fig. 2.

**Table 2:** Superficial retinal thickness measurements of the subjects

Superficial retinal thickness (μm)	Study eyes *N* = 39	Control eyes *N* = 50	P value
	mean ± SD	mean ± SD	
Foveal	37.123 ± 10.515	35.860 ± 10.793	0.587
Parafoveal mean	112.417 ± 7.002	116.680 ± 6.668	0.004
superior	117.564 ± 6.508	121.000 ± 7.273	0.023
temporal	103.308 ± 7.991	107.820 ± 6.675	0.005
inferior	116.359 ± 8.683	122.560 ± 7.859	0.001
nasal	112.436 ± 7.830	115.340 ± 7.061	0.070
Foveal+Parafoveal	97.354 ± 6.770	100.516 ± 6.933	0.034

SD, standard deviation. *P* < 0.05, independent sample t-test

Compared with the control group, the average thickness of parafoveal, the average thickness of parafoveal superior, inferior, temporal quadrants of the superficial retina and the average thickness of the foveal and parafoveal of the superficial retina were significantly decreased in the study group (*P* < 0.05).

#### Deep retinal thickness measurements

**Fig. 3:**
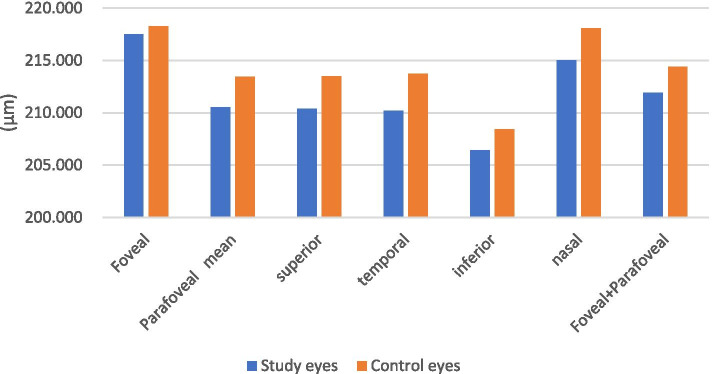
Deep retinal thickness

**Fig. 4:**
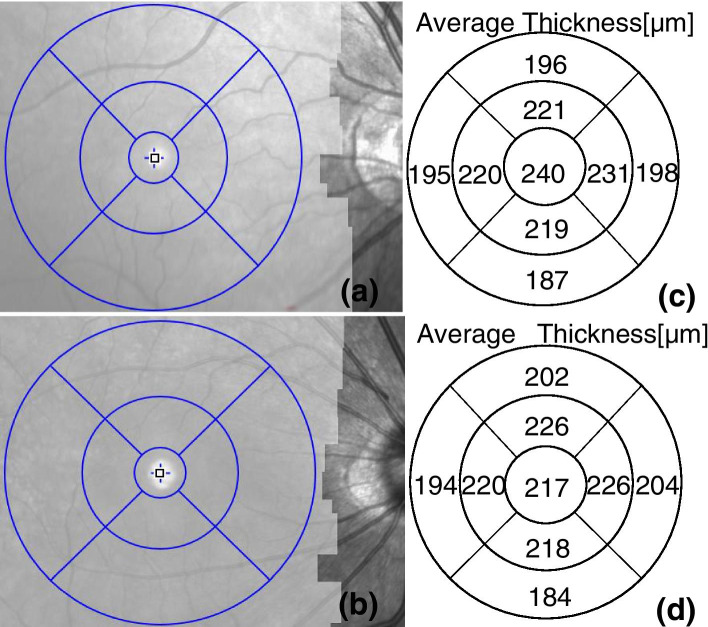
Deep retinal thickness of an study eye (**a**) and a control eye (**b**). (**c**) and (**d**) are the results of each quadrant thickness of deep retina automatically measured by the system software (the numbers in the corresponding area in the figure) respectively

The results of deep retinal thickness in macular region of the study eyes and control eyes are shown in Table 3 and Fig. 3. The representative OCT images are shown in Fig. 4.

**Table 3:** Deep retinal thickness measurements of the subjects

Deep retinal thickness (μm)	Study eyes N = 39	Control eyes N = 50	P value
	mean ± SD	mean ± SD	
Foveal	217.513 ± 11.887	218.260 ± 13.144	0.782
Parafoveal mean	210.513 ± 9.997	213.440 ± 9.212	0.155
superior	210.385 ± 9.751	213.520 ± 9.446	0.129
temporal	210.205 ± 10.519	213.720 ± 8.960	0.093
inferior	206.436 ± 10.382	208.440 ± 9.372	0.342
nasal	215.026 ± 11.451	218.080 ± 10.228	0.188
Foveal+Parafoveal	211.913 ± 9.749	214.404 ± 9.468	0.227

SD, standard deviation. *P* < 0.05, independent sample t-test

The deep retinal thickness of the study eyes was compared with the control eyes. The difference was not statistically significant (P>0.05).

#### Full retinal thickness measurements

**Fig. 5:**
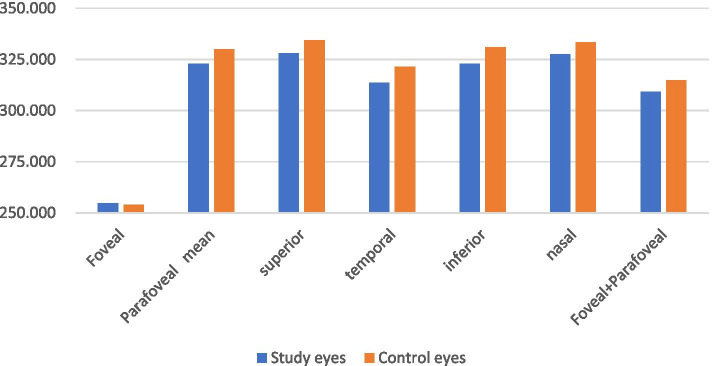
Full retinal thickness

**Fig. 6:**
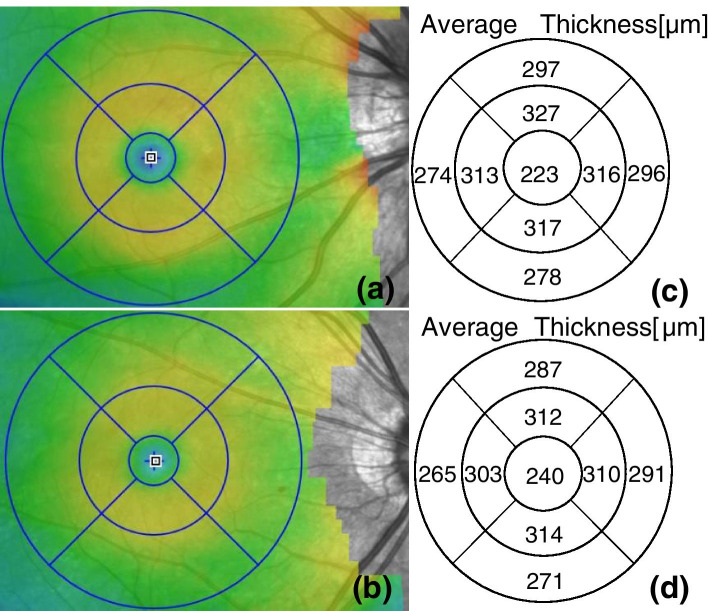
Full retinal thickness of an study eye (**a**) and a control eye (**b**). (**c**) and (**d**) are the results of each quadrant thickness of full retina automatically measured by the system software (the numbers in the corresponding area in the figure) respectively

The results of full retinal thickness in macular region of the study eyes and control eyes are shown in Table 4 and Fig. 5. The representative OCT images are shown in Fig. 6.

**Table 4:** Full retinal thickness measurements of the subjects

Full retinal thickness (μm)	Study eyes *N* = 39	Control eyes N = 50	P value
	mean ± SD	mean ± SD	
Foveal	254.615 ± 19.085	254.120 ± 21.047	0.909
Parafoveal mean	322.930 ± 14.391	330.120 ± 13.682	0.018
superior	327.949 ± 13.700	334.520 ± 14.214	0.031
temporal	313.513 ± 14.164	321.540 ± 13.271	0.007
inferior	322.795 ± 15.732	331.000 ± 14.323	0.012
nasal	327.462 ± 15.456	333.420 ± 14.486	0.065
Foveal+Parafoveal	309.267 ± 14.089	314.920 ± 14.270	0.066

SD, standard deviation. *P* < 0.05, independent sample t-test

Compared with the control group, the average thickness of all quadrants of the retina in the parafoveal area except the nasal quadrant were significantly decreased in the study group (*P* < 0.05).

## Discussion

The results of this study revealed that the average thickness of parafoveal, the average thickness of parafoveal superior, inferior, temporal quadrants of the superficial retina and the average thickness of the foveal and parafoveal of the superficial retina were significantly decreased in the study group compared with the control group. The average thickness of all quadrants of the retina in the parafoveal area except the nasal quadrant were significantly decreased in the study group compared with the control group.

There are few studies on HM pregnant women, and the results of existing studies on retinal thickness of healthy pregnant women are still controversial. In a prospective study of 60 pregnant and 20 non-pregnant women, Cankaya et al. found an increase in total macular volume and foveal retinal thickness in women in the second and third trimester compared to those in the first trimester and non-pregnant women [8]. Demir et al. included 40 women in the third trimester of pregnancy in a prospective study,which found an increase in superior, inferior, temporal parafoveal areas of the macular thickness compared to non-pregnant women [9]. Contradictionally, Ulusoy et al. recruited 29 women in a prospective cohort study in the third trimester of pregnancy and found that there was no difference in macular thickness between the third trimester of pregnancy and non-pregnant women and 3 months postpartum [10]. Atas et al. enrolled 25 women in the third trimester of pregnancy in a prospective study and found that compared to non-pregnant women, the macular thickness decreased in the third trimester of pregnancy [ [6]]. This is similar to the results in our study.

According to the studies, the inconsistency of the research results may be caused by the difference in the measurement area. Cankaya et al. and Demir et al. studied the superior, temporal, inferior and nasal retinal thickness within 1 mm and 3 mm of the macular region. Ulusoy et al. and Atas et al. measured retinal thickness within 1 mm of the macular. In addition, the small number of pregnant women, differences in gestational age and measurement equipment may also have influenced the results.

The changes in hormone levels during pregnancy are one of the fundamental reasons that affect ocular blood flow. The cornea, tarsal gland, lacrimal gland, choroid, retina and other tissues all contain sex hormone receptors. There are sex hormone mRNA expressions in some structures of these tissues [11]. Serum levels of estrogen and progesterone increase throughout pregnancy [12]. These hormones have different effects through their receptors and/or mRNA expression [11, 13]. Estrogen regulates ocular blood flow by regulating vasodilation [14, 15]. Elevated estrogen levels will lead to an increase in the synthesis of nitrous oxide and a decrease in the synthesis of endothelin-1, both of which will lead to a decrease in vasodilation and vascular resistance [16]. As an estrogen antagonist, progesterone can cause vasoconstriction. However, estrogen has a major impact on the human circulatory system [17]. Centofanti et al. found that pregnant women had higher ocular blood flow, which may be due to the endothelium-dependent vasodilation effect of estrogen [18]. Similarly, Sato et al. used laser speckle imaging technology demonstrated a reduction in retinal vascular resistance during pregnancy [19].

Another reason that affects the ocular vascular system during pregnancy is the changes in the cardiovascular system, which cater to the increased metabolic demands of the mother and fetus. It mainly includes changes in heart rate, systemic vascular resistance and blood volume [20]. During pregnancy, systemic vascular resistance decreases and blood volume increases [21, 22]. Systolic and diastolic blood pressure usually drop by 5-10 mmHg. The drop in blood pressure is caused by a decrease in systemic vascular resistance [23]. The above changes in the cardiovascular system can increase the blood flow to different organs such as the kidneys, brain and uterus [24–26]. However, the blood flow may be redistributed to other important organs (such as the uterus and kidneys) and skin, resulting in a relative decrease in ocular blood flow in the third trimester of pregnancy [27]. Therefore, the effect of HM and pregnancy on retinal thickness and its underlying mechanism are still unclear. Further studies are needed to confirm.

Our study has the following limitations. First of all, the sample size of this study was small and it was a single-center study. Considering the safety of mothers and infants, and the acceptance of the subjects, only the relatively safe third trimester are selected for observation. There was no follow-up to observe the dynamic changes of retinal thickness in pregnant women from early pregnancy to postpartum. In the future, more large-scale longitudinal studies are needed to confirm the effect of pregnancy on retinal blood flow and structure in HM patients.

## Conclusions

In conclusion, the retinal thickness of patients with high myopia during the third trimester of pregnancy was thinner than that of non-pregnant women with age-matched high myopia. Further studies are needed to determine the potential mechanisms and clinical relevance of this finding.

## Data Availability

The data that support the findings of this study are available from the corresponding author on reasonable request.

## Abbreviations

AL: Axial length
BCVA: best corrected visual acuity
HM: High myopia
ILM: internal limiting membrane
INL: inner nuclear layer
IOP: Intraocular pressure
IPL: inner plexiform layer
LogMAR: logarithm of the minimum angle of resolution
OCT: optical coherence tomography
RPE: Retinal pigment epithelium
SD: Standard deviation
